# Acetylshikonin induces apoptosis through the endoplasmic reticulum stress‐activated PERK/eIF_2α_/CHOP axis in oesophageal squamous cell carcinoma

**DOI:** 10.1111/jcmm.18030

**Published:** 2023-11-06

**Authors:** Ya‐Jiao Yuan, Shanshan Liu, Hong Yang, Jian‐Ling Xu, Jing Zhai, Han‐Ming Jiang, Beibei Sun

**Affiliations:** ^1^ Department of Biochemistry and Molecular Biology, College of Clinical and Basic Medicine Shandong First Medical University & Shandong academy of medical sciences Jinan China; ^2^ Department of Clinical Laboratory Qingdao Jimo People's Hospital Qingdao China; ^3^ Department of Clinical Laboratory Taian Central Hospital China

**Keywords:** acetylshikonin, apoptosis, endoplasmic reticulum stress, oesophageal squamous cell carcinoma

## Abstract

Acetylshikonin (AS) is an active component of *Lithospermum erythrorhizon* Sieb. et Zucc that exhibits activity against various cancers; however, the underlying mechanisms of AS against oesophageal squamous carcinoma (ESCC) need to be elusive. The research explores the anti‐cancer role and potential mechanism of AS on ESCC in vitro and in vivo, providing evidences for AS treatment against ESCC. In this study, we firstly demonstrated that AS treatment effectively inhibits cell viability and proliferation of ESCC cells. In addition, AS significantly induces G1/S phage arrest and promotes apoptosis in ESCC cell lines. Further studies reveal that AS induces ER stress, as observed by dose‐ and time‐dependently increased expression of BIP, PDI, PERK, phosphorylation of eIF_2α_, CHOP and splicing of XBP1. CHOP knockdown or PERK inhibition markedly rescue cell apoptosis induced by AS. Moreover, AS treatment significantly inhibits ESCC xenograft growth in nude mice. Elevated expression of BIP and CHOP is also observed in xenograft tumours. Taken together, AS inhibits proliferation and induces apoptosis through ER stress‐activated PERK/eIF_2α_/CHOP pathway in ESCC, which indicates AS represents a promising candidate for ESCC treatment.

## 1 INTRODUCTION

Oesophageal carcinoma (EC) is one of the most frequent upper gastrointestinal malignancy, which ranks 8th in incidence and 6th in mortality globally.^1^ In 2020, there were an estimated 604,100 new cases of oesophageal cancer in the whole world. Of all cases, 4.6% occurred in Africa, 6.3% occurred in America, 8.9% occurred in Europe and 79.7% occurred in Asia. Among them, 59.2% occurred in Eastern Asia and 53.7% occurred in China alone.^1^ The main histological categories are adenocarcinoma (EAC) and squamous cell carcinoma (ESCC).^2^ Currently, China has emerged as the country with the highest incidence of EC, and >90% of EC patients are diagnosed with ESCC.^3^ Endoscopic resection combined with radiotherapy and chemotherapy represent the standard treatment for ESCC^4^, ^5^; however, outcomes remain unsatisfactory because of the high recurrence rate and the development of drug and radiation resistance.^6^ The 5‐year survival rate of patients with ESCC is less than 30%.^7^ Therefore, it is of importance to explore novel chemotherapeutic agents to treat ESCC.

The endoplasmic reticulum (ER) as a specialized organelle involves in protein synthesis, folding and transportation through dynamic structural changes^8^; thus, the maintenance and homeostasis of the ER environment are necessary. Various endogenous or exogenous stimuli may damage the morphology and function of the ER (ER stress), which promotes the misfolding of proteins and subsequently primes the cell to respond and restore function by activation of unfolded protein response (UPR).^9^ The activated UPR helps protein fold properly and removes misfolded proteins to maintain ER homeostasis.^10^ However, sustained ER stress may cause the cell to switch from a pro‐survival response to a pro‐death mode, which results in apoptotic or autophagic cell death.^11^ There are three signalling pathways related to the UPR, protein kinase RNA‐dependent‐like ER kinase (PERK), inositol‐requiring protein‐1 (IRE1) and activating transcription factor 6 (ATF6). Sustained ER stress induces death signals through each of these ways.^12^, ^13^ Thus, identifying small molecules that trigger apoptosis mediated by ER stress represents a novel strategy for cancer therapy.

Acetylshikonin (AS) is a fat‐soluble naphthoquinone pigment that exhibits broad bioactivity, such as anti‐tumour, anti‐inflammation and antibacterial activity.^14^ Among these, the anti‐tumour properties have been well‐described.^15^, ^16^, ^17^ AS predominantly suppresses tumour progression by triggering apoptosis in various cancer cells.^18^, ^19^, ^20^, ^21^, ^22^, ^23^ But the anti‐cancer effects and mechanisms of AS in ESCC have not been defined. It was reported that AS induces apoptosis via ER stress in human hepatocellular carcinoma cells.^24^ We demonstrated that AS treatment triggered sustained ER stress and activated the PERK/eIF_2α_ pathway, resulting in up‐regulation of CHOP. Both inhibition of PERK and siRNA‐mediated knockdown of CHOP rescued cells from AS‐induced apoptosis. Our findings indicate that AS is an attractive candidate for further development as ESCC therapy.

## 2 MATERIALS AND METHODS

### 2.1 Compounds and reagents

The extraction, separation and purification of AS have been described previously.^25^ AS was prepared to 10 mM with DMSO and dissolved in culture medium prior to use. The Cell Counting Kit‐8 (CCK‐8) (Cat No. C0037) was obtained from Beyotime Institute of Biotechnology. The apoptosis assay kit (Cat No. BD556547) was from BD Biosciences. The Giemsa staining (Cat No. G1015) and BCA protein assay kits (Cat No. PC0020) were purchased from Solarbio Life Sciences. GSK2606414 (Cat No. S7307), a PERK inhibitor, was obtained from Selleck. Protease inhibitor cocktail (Cat No. FD1001) and protein phosphatase inhibitors (Cat No. FD1002) were purchased from FUDE Biological Technology. Antibodies against cleaved PARP (Cat No. 5625), active caspase 3 (Cat No. 9664), BAX (Cat No. 41162), BIP (Cat No. 3177), PDI (Cat No. 3501), calnexin (Cat No. 2679), CHOP (Cat No. 2895) and ERO1‐Lα (Cat No. 3264) were bought from CST company. Antibody against GAPDH (Cat No. sc‐47724) was obtained from Santa Cruz Technology. All the antibodies used in immunohistochemistry (Cat No. GB11098‐100, GB111141‐100, GB23303) were provided by Servicebio. Peroxidase‐labelled secondary antibodies against mouse (Cat No. 074‐1806) and rabbit (Cat No. WBKlS0100) (Cat No. 074‐1506) were provided by KPL. Enhanced chemiluminescence (ECL) detection kit (Cat No. WBKlS0100) was obtained from Millipore. The Kit (Cat No. R6934‐02) used for RNA extraction was from Omega. RT‐PCR kit (Cat No. R211‐01) and qPCR assay kit (Cat No. R122‐01) were bought from Vazyme.

### 2.2 Cell lines and culture

Six cell lines of ESCC were purchased from the National Collection of authenticated cell cultures. Cell culture has been described previously.^26^ In brief, KYSE450 and TE10 cells were cultured in MEM and DMEM, respectively, with 10% FBS and 1% antibiotic mix. KYSE180, KYSE510, EC109 and EC9706 cells were maintained in RPMI‐1640 media supplied with 10% FBS and 1% antibiotic mix.

### 2.3 Cell viability assay

The KYSE450 cells were grown in 96‐well plates at 4 × 10^3^ cells/well, while the other cell lines were seeded at 5 × 10^3^ cells/well. After adhesion, all cells were treated with DMSO or AS for 24 h and then subjected to CCK‐8 assay.^26^ IC_50_ values were determined by GraphPad Prism 8.0. The results were from three independent experiments.

### 2.4 Clonogenicity assay

The clonogenicity assay was described previously with minor modifications.^27^ Briefly, the KYSE180 cells (1 × 10^3^ cells per well) were incubated with AS for 2 weeks. Then the cold methanol was added to fix the cells. After washing with PBS, the colonies were visualized and counted after staining with 10% Giemsa dye.

### 2.5 Cell apoptosis analysis

KYSE180 and KYSE450 cells were cultured in 6‐well plate overnight and then incubated with AS for 24 h. The cells were collected to investigate apoptosis by flow cytometer (BD FACS Canto II) using the apoptosis assay kit. For combination assays, after transfecting with siRNAs for 24 h or pretreating with PERK inhibitor for 2 h, KYSE180 and KYSE450 were treated with AS for 24 h.

### 2.6 Cell cycle assay

The KYSE450 cells were seeded and treated as indicated. The cells were collected and suspended with cold ethanol for fixation. After stained with propidium iodide (PI), the distribution of cell cycle was analysed by flow cytometer.

### 2.7 Western blot assay

The treated cells were collected, and total proteins were prepared by SDS buffer containing protease inhibitors. After centrifugation, the supernatants were collected and quantified by the BCA assay. The 20 μg protein were resolved by SDS‐PAGE, transferred to nitrocellulose membranes. After blocking, the membranes were incubated with primary antibody and secondary antibody, respectively. Finally, immunoreactive bands were shown after detecting by the ECL reagent. GAPDH served as control for normalizing the amount of protein.

### 2.8 Quantitative RT‐PCR

Total RNA extraction and RT‐PCR were performed previously.^26^ Quantitative PCR was performed using ChamQTM SYBR® qPCR kit on a Mx3005P PCR instrument (Agilent). The specific primers are as follows: ATF3, forward: 5′‐TAG CCC CTG AAG AAG ATG AAA G‐3′ and reverse: 5′‐CTT CTT CTT GTT TCG GCA CTT T‐3′; ATF4, forward: 5′‐ATG GAT TTG AAG GAG TTC GAC T‐3′ and reverse: 5′‐AGA GAT CAC AAG TGT CAT CCA A‐3′; ATF6, forward: 5′‐CTG ATG GCT GTT CAA TAC ACA G‐3′ and reverse: 5′‐GAT CCC TTC GAA ATG ACA CAA C‐3′. The primers of BIP, CHOP and GAPDH used in this study were described previously.^28^, ^29^, ^30^ GAPDH served as control to normalize mRNA expression level.

### 2.9 XBP1 splicing analysis

After AS treatment, total RNA was harvested and reversely transcribed for XBP1 splicing analysis. Specific primers for XBP1 were used to amplify XBP1 transcripts by RT‐PCR. The products were digested with *Pst* I as reported previously.^21^ After digestion, the fragments were applied to the 2% agarose gel to identify the spliced and unspliced products of XBP1.

### 2.10 SiRNA Transfection

The sequences of CHOP siRNA were described previously.^31^ A scramble siRNA was used as control. The KYSE180 and KYSE450 cells were incubated with 10 nm of scramble siRNA or siRNA duplex (OBiO Technology) using INTERFERin (Polyplus) according to the protocol. The transfected cells were exposed to AS for 24 h.

### 2.11 Mouse xenograft assay

Animal studies were approved by the animal ethics committee of Shandong First Medical University. Thirty athymic BALB/c nude mice were housed in SPF level of animal house with the 12‐h light and 12‐h dark cycle. 100 μl suspension containing 5 × 10^6^ cells of KYSE180 was implanted subcutaneously into the right flank of a mouse. All mice were randomly classified into three groups, the control group, low‐dose group and high‐dose group, until the tumour volume reached approximately 100 mm^3^. Tumour size was determined with Vernier callipers. The formula was used to calculate tumour volume is that V = (L × W^2^)/2, where V means tumour volume, L represents length and W is width. The control group mice were treated with a vehicle solution (0.3% 2‐hydroxypropyl‐beta cyclodextrin). The low‐dose group and high‐dose group mice were intraperitoneally injected with 10 mg/kg AS and 20 mg/kg AS, respectively. All mice were treated with AS every 2 days. The body weight and tumour volumes were monitored every 2 days. After 3 weeks, all mice were sacrificed. The tumours were collected for weighing and immunohistochemistry analysis.

### 2.12 Immunohistochemistry

The fixed and paraffin‐embedded samples were sectioned into 5 μm slices. The specimens were dewaxed in xylene and then rehydrated by gradually decreasing concentrations of ethanol (100%–75%). After washed, the slides were heated in 0.01 M citrate buffer (pH 6.0) for antigen retrieval. After spontaneous cooling, the samples were rinsed with PBS (pH 7.4) thrice on a shaking table, treated with 3% H_2_O_2_ in the dark at regular temperature for 25 min and subsequently rinsed with PBS (pH 7.4) thrice. The samples were blocked with 3% BSA for 30 min and incubated with primary antibodies overnight at 4°C in a humidified chamber. Ki67 (1:500), CHOP (1:1000) and BIP (1:800) antibodies were used. After washing, the samples are incubated with secondary antibody labelled with HRP and DAB, the slides were counterstained with haematoxylin and images were captured. The tumour specimens were also analysed by H&E staining.

### 2.13 Data statistical analysis

The results were analysed by GraphPad Prism 8.00 and displayed as the mean ± SD. Two‐tailed Student's *t*‐test was used to determine the significance between two groups. The *p* < 0.05 was set as statistical significance.

## 3 RESULTS

### 3.1 AS inhibited the viability, proliferation and cell cycle progression in ESCC cells

**FIGURE 1:**
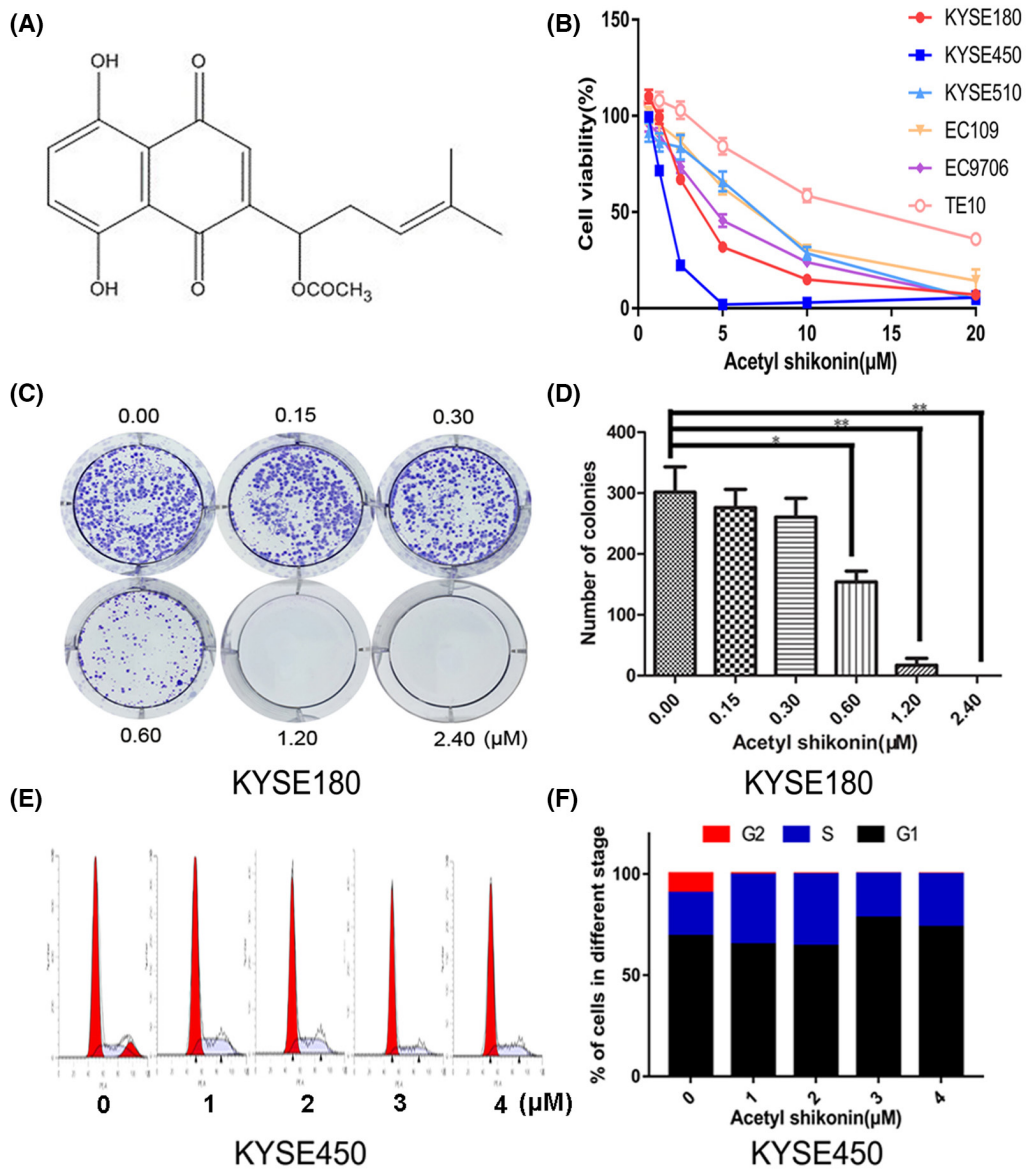
Acetylshikonin (AS) inhibited viability and proliferation of ESCC cells. (A) The chemical structure of AS. (B) AS decreased ESCC cells viability in a dose‐dependent manner. The cell viability of six ESCC cell lines was estimated by CCK8 assay after treating with AS for 24 h. (C) AS inhibited clonogenicity of KYSE180 cells in a dose‐dependent manner. (D) The numbers of colonies were shown in the statistical chart. *p < 0.05, **p < 0.01. Data were presented as mean ± SD of three independent experiments. (E) AS‐induced cell cycle arrest in KYSE450 cells. (F) After AS treatment, KYSE450 cells exhibited S phase arrest at low concentrations and G1 phase arrest at medium to high concentrations.

To determine the anticancer effect of AS (Figure 1A) on ESCC cells, the KYSE450, KYSE180, KYSE510, TE10, EC109 and EC9706 cells were treated with increasing dosages of AS for 24 h. Cell viability was assessed by the CCK8 assay. As shown in Figure 1B, the viability of all cell lines was significantly decreased following AS treatment. The IC_50_ values varied from 1.69 to 13.25 μΜ (Table 1). Among which, KYSE180 and KYSE450 were more sensitive to AS as evidenced by lower IC_50_ values. We also found that AS dose‐dependently inhibited the clonogenicity of KYSE180 cells (Figure 1C). The KYSE180 cells could hardly develop a colony when the AS concentration was greater than 1.2 μΜ (Figure 1D).

**TABLE 1:** IC50 of acetylshikonin in six cell lines of ESCC.

Cell lines	IC50 (μΜ)
KYSE450	1.69
KYSE180	3.72
KYSE510	6.29
EC109	6.66
EC9706	4.60
TE10	13.25

To confirm whether the AS‐induced proliferation inhibition due to cell cycle arrest, the KYSE450 cells were exposed to AS and then the proportion of cell phases was analysed by flow cytometer. The results indicated that AS significantly blocked cell cycle at G1/S phase as evidenced by increasing proportion of G1/S phase (Figure 1E). Interestingly, low dosages (<2 μΜ) of AS significantly exhibited S phase arrest. When AS concentration was more than 2 μΜ, the cell cycle was blocked at G1 phase in KYSE450 cells (Figure 1F).

Taken together, AS suppressed the viability and proliferation in ESCC cells and blocked cell cycle progression. AS may be a potent cytotoxic agent for ESCC treatment.

### 3.2 AS‐induced apoptosis in KYSE180 and KYSE450 cells

**FIGURE 2:**
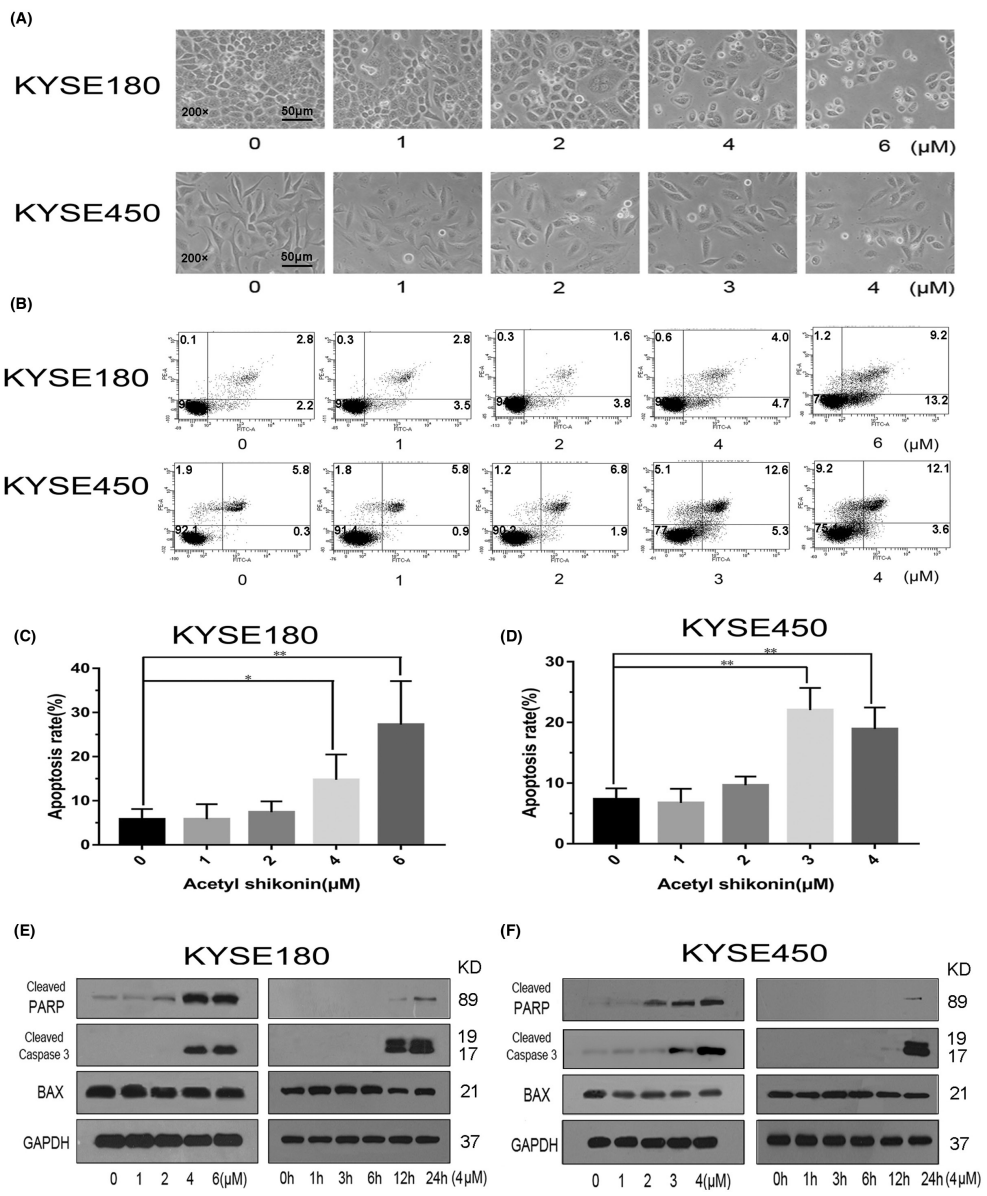
Acetylshikonin (AS) induced apoptosis in KYSE180 and KYSE450 cells. (A) The morphology of KYSE180 and KYSE450 cells changed with AS treatment. The magnification was 200× and the scale bar represented 50 μm. (B) AS dramatically increased Annexin V‐positive cells in KYSE180 and KYSE450 cells. The cells were treated with indicated AS for 24 h, stained with Annexin V/PI and analysed by flow cytometry. The percentages of Annexin V‐positive cells were shown. (C, D) The quantitative data of panel. The data were displayed as the mean ± SD (*n* = 3). *p < 0.05, **p < 0.01. (E, F) KYSE180 and KYSE450 cells were treated with different concentrations of AS for 24 h or treated with 4 μM of AS for different periods. The expression levels of apoptosis‐related proteins were detected by WB. GAPDH was used as an internal control.

To determine whether AS‐triggered apoptosis in ESCC cells, the KYSE180 and KYSE450 cells were incubated with AS for 24 h. The results demonstrated that AS‐induced morphological characteristics of apoptosis in the two cell lines in a dose‐dependent pattern (Figure 2A). We further examined the apoptotic rate by flow cytometry. AS sharply triggered apoptosis in KYSE180 and KYSE450 cells due to the increased proportion of FITC‐positive cells (Figure 2B). The apoptotic rate reached 28.6% when treated with 6 μM AS in KYSE180 cells (Figure 2C). For KYSE450 cells, the apoptotic rate was 22.8% following 3 μM AS treatment (Figure 2D). We further investigated the changes of apoptosis‐related proteins by WB. We found the levels of cleaved PARP and caspase 3 were no difference when the AS was below 2 μM. However, cleaved PARP and caspase 3 were significantly increased when the AS was more than 4 μM. In addition, the levels of cleaved PARP and caspase 3 were dramatically increased when AS exposure was more than 12 h (Figure 2E,F). However, Bax levels were not increased by AS. These data indicated that AS dose‐ and time‐dependently triggered apoptosis by a caspase‐dependent manner in KYSE180 and KYSE450 cells.

### 3.3 AS‐triggered apoptosis by activating the ER stress pathway

**FIGURE 3:**
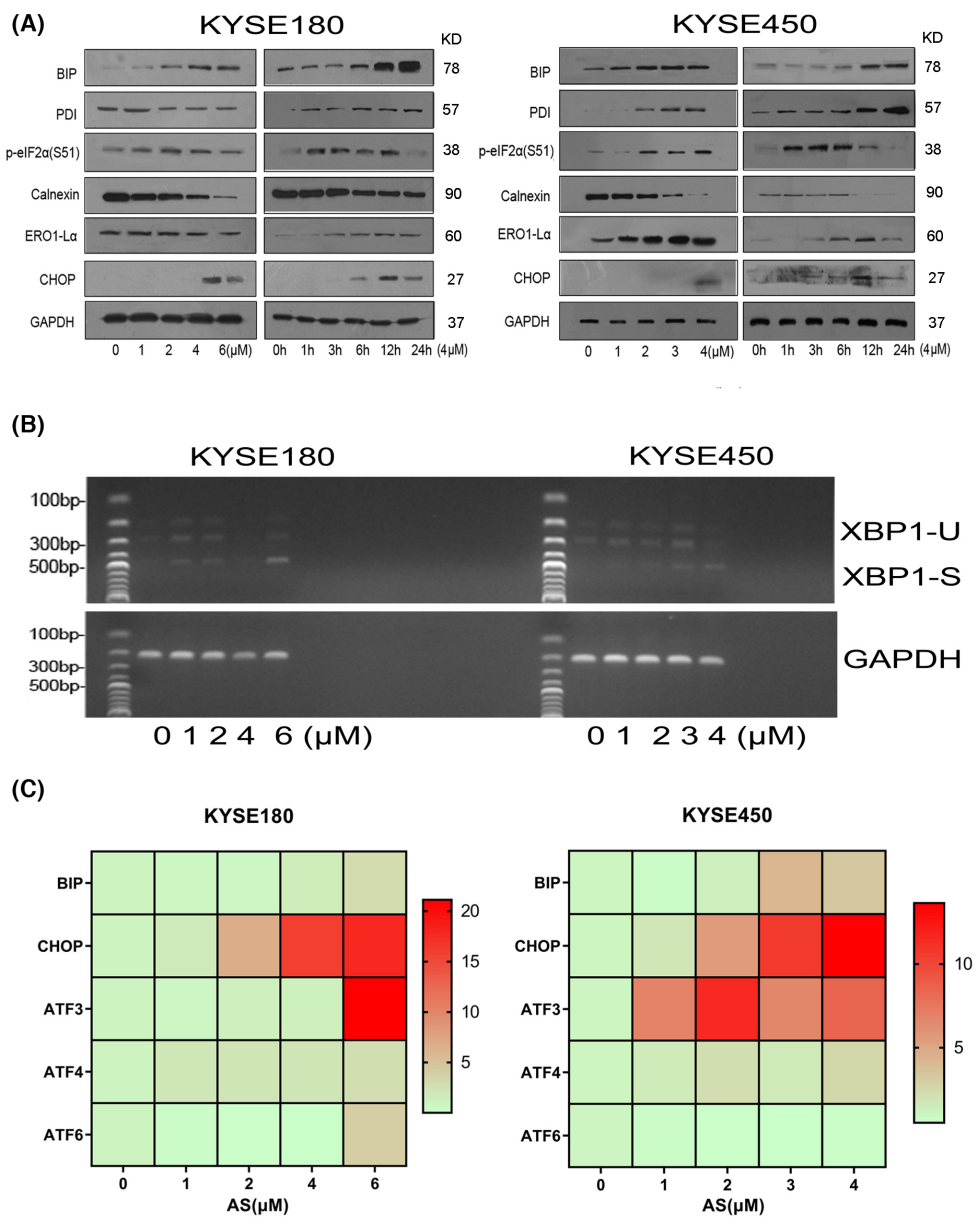
Acetylshikonin (AS) induced apoptosis through activating the ER stress pathway. (A) KYSE180 and KYSE450 cells were treated with different concentrations of AS for 24 h or with the 4 μM of AS for different periods, and WB was performed to detect the expression levels of proteins related to ER stress pathway. GAPDH‐normalized quantitative data are shown below the panel. (B) AS increased the shear of IRE1 to XBP1. (S: sheared XBP1 fragments; U: unsheared XBP1 fragments). (C) AS significantly increased the mRNA levels of ER stress‐related gene in a dose‐dependent manner.

It was reported that natural naphthoquinones triggered apoptosis through activation of the ER stress pathway in numerous cancers.^32^, ^33^ To determine whether ER stress was induced by AS treatment, we examined several well‐known proteins associated with ER stress following AS treatment. BIP, PDI and Ero1‐Lα were time‐dependently up‐regulated with AS treatment in the two ESCC cell lines (Figure 3A). In addition, eIF_2α_ phosphorylation increased markedly at 6 h and maintained an elevated level up to 24 h; however, calnexin was significantly decreased. We also observed that CHOP, an ER stress‐associated pro‐apoptotic protein, exhibited the similar observation to eIF_2α_ phosphorylation.

To further confirm AS‐induced ER stress, we examined the splicing of the X‐box‐binding protein 1 (XBP1) in AS‐treated cells. It was shown that the spliced XBP1 mRNA was dose‐dependently up‐regulated in responding to AS administration, which indicated that the IRE1/XBP1 axis was activated in KYSE180 and KYSE450 cells (Figure 3B). Next, we analysed gene expression levels by qRT‐PCR in AS‐treated cells. The results indicated that AS markedly induced BIP, CHOP, activating transcription factor 3 (ATF3) and activating transcription factor 4 (ATF4) at the transcriptional level. Meanwhile, the activating transcription factor 6 (ATF6) mRNA levels were only slightly up‐regulated with AS administration (Figure 3C). Taken together, AS‐induced sustained ER stress and activated PERK/eIF_2α_ and IRE1/XBP1 signalling.

**FIGURE 4:**
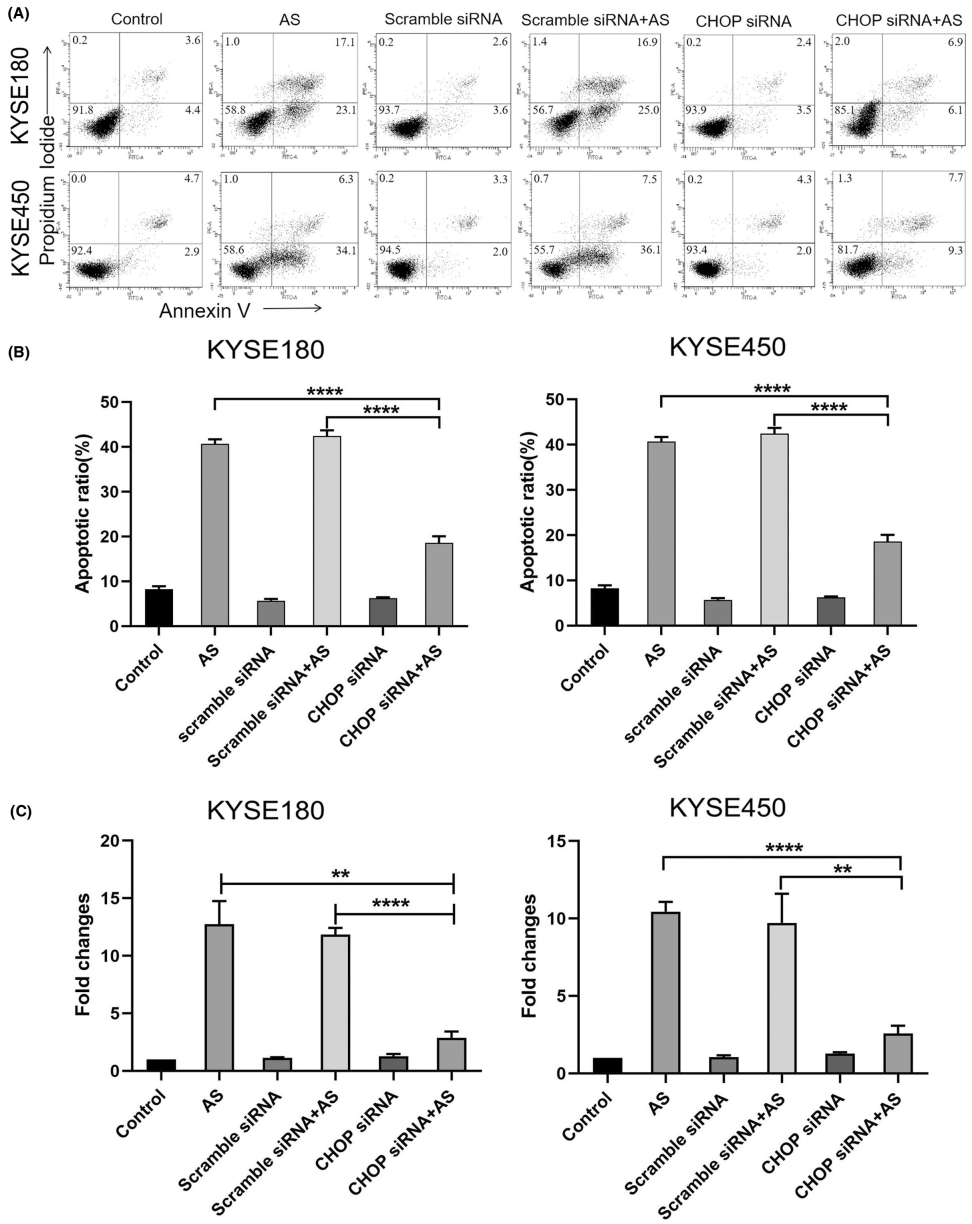
Acetylshikonin (AS) induced apoptosis through up‐regulation of CHOP in KYSE180 and KYSE450 cells. (A) Knockdown of CHOP dramatically blocked AS‐induced apoptosis in KYSE180 and KYSE450 cells, as tested by Annexin V and PI staining and analysed by flow cytometry. The percentages of Annexin V‐positive cells were shown. (B) The quantitative data of panel. The data were displayed as the mean ± SD (*n* = 3). ****p < 0.0001. (C) The efficacy of CHOP siRNA by real‐time PCR. The data were analysed by graphpad prism 8 and displayed as the mean ± SD (*n* = 3). **p < 0.01, ****p < 0.0001.

**FIGURE 5:**
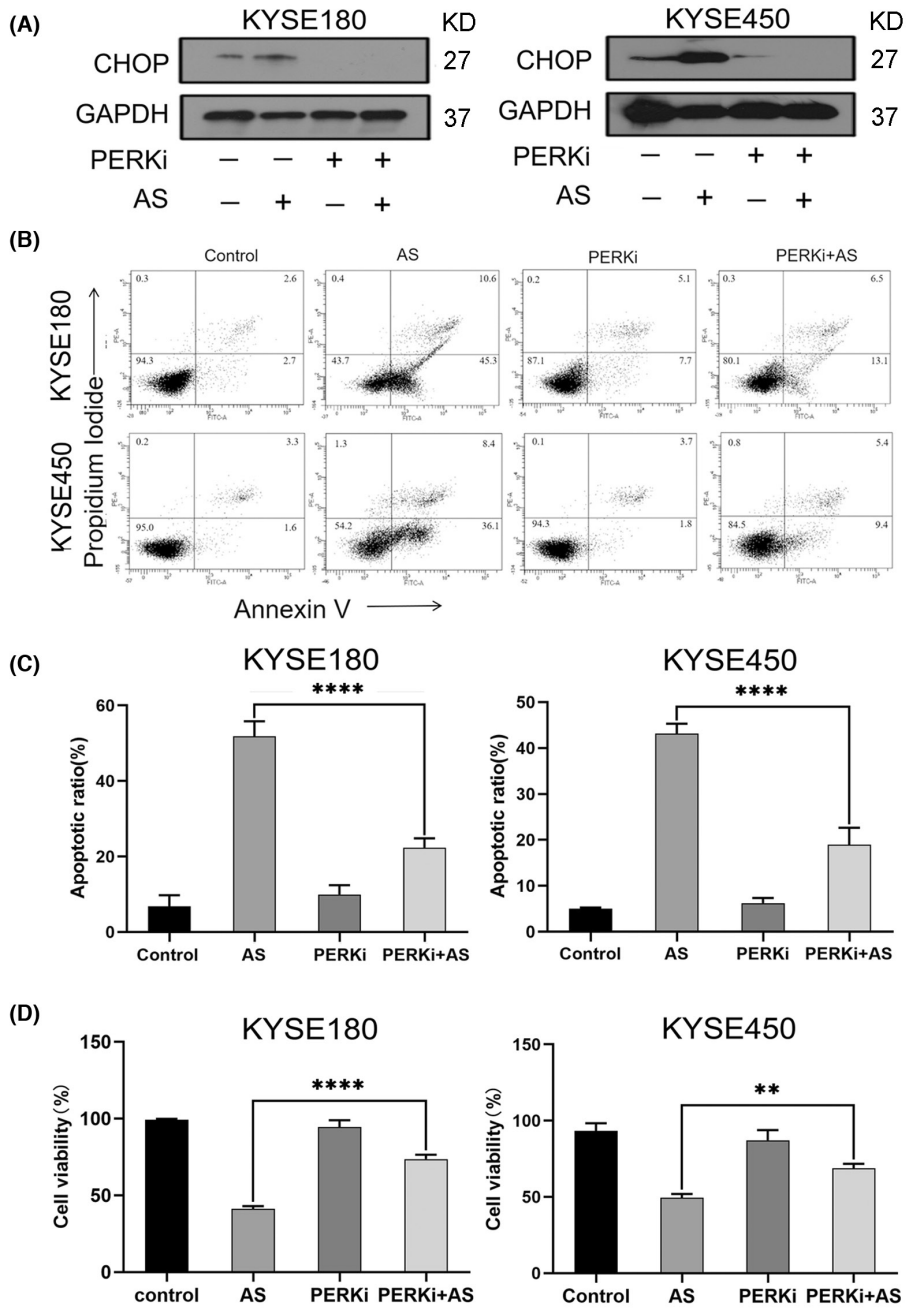
Acetylshikonin (AS) induced apoptosis through activated PERK/eIF_2α_/CHOP axis in KYSE180 and KYSE450 cells. (A) The down‐regulation of CHOP in the presence of GSK2606414 (PERKi), as tested by WB. GAPDH‐normalized quantitative data are shown. (B) Inhibition of PERK dramatically blocked AS‐induced apoptosis in KYSE180 and KYSE450 cells, as tested by Annexin V and PI staining and analysed by flow cytometry. The percentages of Annexin V‐positive cells were shown. (C) The quantitative data of panel. The data were displayed as the mean ± SD (*n* = 3). ****p < 0.0001. (D) The cell viability inhibited by AS was significantly restored in the presence of GSK2606414 (PERKi).

Sustained ER stress activates CHOP‐induced apoptosis.^34^, ^35^, ^36^, ^37^, ^38^ To determine whether AS‐triggered apoptosis in ESCC cells through the up‐regulation of CHOP, we knocked down CHOP with siRNA and measured apoptosis in KYSE180 and KYSE450 cells. We found that AS‐triggered apoptosis was markedly reduced in the absence of CHOP as evidenced by a reduction of FITC‐positive cells (Figure 4A,C). The apoptotic rate decreased from 38.8% to 5.8% following treatment with 6 μM AS in KYSE180 cells (Figure 4B). For KYSE450 cells, the apoptotic rate decreased from 40.7% to 6.23% following 4 μM AS treatment (Figure 4B). Together, these observations indicated that CHOP played critical role in AS‐induced apoptosis. Activation of PERK/eIF_2α_ cascade is associated with CHOP expression in response to ER stress.^39^, ^40^, ^41^ To characterize the function of the PERK/eIF_2α_ axis in AS‐triggered CHOP up‐regulation, we pretreated the KYSE180 and KYSE450 cell lines with GSK2606414, a PERK inhibitor for 2 h to inhibit PERK activity, and assessed CHOP expression and apoptosis induced by AS treatment. The results demonstrated that GSK2606414 alone scarcely affected cell viability, whereas AS‐induced CHOP was markedly decreased by GSK2606414 (Figure 5A,D). Consistently, cell viability was significantly restored and apoptosis decreased after GSK2606414 treatment (Figure 5B–D). These data indicated that the AS‐activated PERK/eIF_2α_ pathway up‐regulated CHOP expression, leading to apoptosis in KYSE180 and KYSE450 cells.

### 3.4 AS delayed ESCC growth in nude mice

**FIGURE 6:**
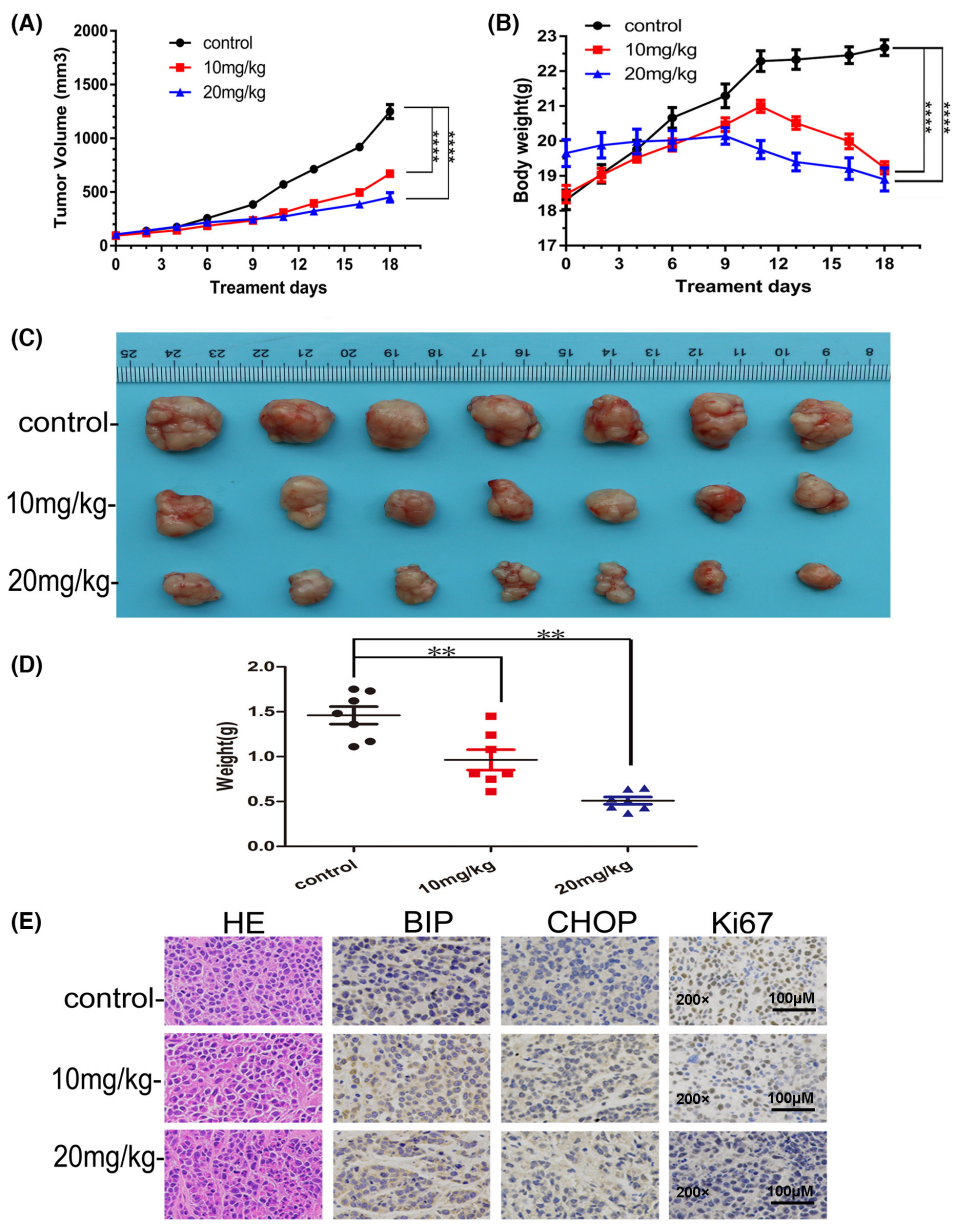
Acetylshikonin (AS) significantly inhibited the growth of KYSE180 tumour xenografts in nude mice. (A) Over the course of 3 weeks, AS significantly suppressed the growth of KYSE180 tumour xenografts compared to the control. (B) AS decreased body weight of mice. The data were displayed as the mean ± SD (*n* = 3). ****p < 0.0001. (C) Photographs of the tumours collected after 3 weeks of treatment with vehicle or AS. (D) AS dramatically decreased tumour weights. Data are presented (*n* = 7). **p < 0.01. (E) Representative immunohistochemistry staining images of BIP, CHOP and Ki67. The assay was performed on these three groups of tumour samples. The magnification was 200×, and the scale bar represented 100 μm.

Since AS markedly suppressed the viability and proliferation of ESCC cells, we determined its potential to inhibit ESCC tumour growth. We successfully implanted KYSE180 cells into nude mice to establish a tumour xenograft model. After treatment of AS, we found AS dose‐dependently inhibited the growth of KYSE180 xenografts as evidenced by the smaller tumour volume (Figure 6A,C). Analysis of tumours indicated that AS dose‐dependently decreased tumour weight and volume compared with the control (Figure 6D); however, the body weights decreased after 3 weeks (Figure 6B). Further immunohistochemistry analysis confirmed that AS inhibited ESCC growth as proved by a dramatical reduction in Ki67‐positive cells (Figure 6E). Furthermore, expression level of BIP and CHOP also increased in xenograft tumours, which indicated AS‐induced ER stress in vivo (Figure 6E).

## 4 DISCUSSION

Natural small molecules derived from traditional Chinese medicine are valuable resources for the development of anti‐tumour drugs because of their novel structure and diverse bioactivites. *Lithospermum erythrorhizon*, also called ‘Zicao’ is an herbal plant that has been used traditionally to treat inflammation, infection, burns and carbuncles in China.^42^ Recently, many studies have revealed that Zicao possesses multiple chemical ingredients and exhibits wound healing, anti‐cancer and anti‐inflammatory activities. Shikonin, the main active constituents of Zicao, has been proved that exerts cytotoxic effects on cancer cells.^42^, ^43^, ^44^ AS is a derivative of shikonin, and its pharmacological effects, particularly its anti‐cancer effect, has attracted significant attention^45^, ^46^; however, it is unclear whether AS exhibits a growth‐inhibitory effect on ESCC. In the present study, we found that AS exerted anticancer activity in ESCC cells as proved by lower IC_50_ values. AS not only effectively decreased cell viability and proliferation, but also inhibited cell cycle progression in ESCC cell lines. Furthermore, AS‐induced apoptosis in ESCC cell lines. Mechanistically, we demonstrated that AS triggers apoptosis mediated by ER stress‐activated PERK/eIF_2α_/CHOP pathway. CHOP knockdown and PERK inhibition significantly rescued AS‐induced apoptosis. Overall, our findings indicate that AS exerts anticancer potential in ESCC, in part, through apoptosis mediated by the ER stress pathway.

The ER is the most important organelle that participate in the synthesis and folding of protein, stress‐sensing and calcium storage.^47^ Cellular stress, including oxidative injury, hypoxia, calcium depletion and viral infection, impairs ER homeostasis, leading to the accumulation of misfolded and unfolded proteins, which activates URP and evokes ER stress.^48^, ^49^ The UPR works through three signal sensors, PERK, ATF6 and IRE1α. They are normally inactive and interact with the chaperone GRP78/BIP.^50^ These molecules are activated by dissociation from BIP when ER stress occurs.^47^ Activated PERK selectively phosphorylates and inactivates eIF_2α_, which causes global translation inhibition and improves the translation of ATF4 and CHOP.^34^ The active IRE1α splices an intron into XBP1 mRNA, converting it from an unspliced form to a spliced form. The spliced XBP1 is translated into the XBP1 protein, which regulates the expression of many UPR‐responsive genes.^51^ Generally, ER stress plays a cytoprotective role, but severe or sustained ER stress induces apoptosis through various pathways. Here, we found that AS‐triggered sustained ER stress and activated PERK and IRE1α, resulting in eIF_2α_ phosphorylation and XBP1 splicing. In the absence of eIF_2α_ activity, the ATF4 expression was markedly up‐regulated, which sequentially enhanced CHOP expression and induced apoptosis. CHOP knockdown markedly reduced apoptosis induced by AS. Therefore, AS may trigger apoptosis through the ER stress‐activated PERK‐eIF_2α_/CHOP axis; however, AS also activates IRE1α and ATF3. Previous studies showed that IRE1α and ATF3 may be involved in apoptosis.^52^, ^53^, ^54^, ^55^, ^56^ We could not exclude whether IRE1α and ATF3 participate in apoptosis triggered by AS in ESCC cells which need further exploration. Furthermore, we also demonstrated that AS treatment significantly reduced tumour growth in nude mice and triggered ER stress.

In conclusion, our research indicated that AS inhibited ESCC cell proliferation and triggered apoptosis. AS effectively induced sustained ER stress and promoted apoptosis via the PERK/eIF_2α_/CHOP cascade. We proved that AS might be a promising candidate for ESCC treatment.

## AUTHOR CONTRIBUTIONS

**Ya‐Jiao Yuan:** Data curation (equal); investigation (equal); methodology (equal); project administration (equal); validation (equal); writing – original draft (equal). **Shanshan Liu:** Data curation (equal); methodology (equal); validation (equal). **Hong Yang:** Data curation (equal); methodology (equal); validation (equal). **Jian‐Ling Xu:** Methodology (supporting); validation (supporting). **Jing Zhai:** Project administration (supporting); supervision (supporting). **Han‐Ming Jiang:** Funding acquisition (supporting); supervision (lead); writing – review and editing (lead). **Beibei Sun:** Funding acquisition (lead); project administration (lead); supervision (lead); writing – review and editing (lead).

## FUNDING INFORMATION

This study was supported by the National Natural Science Foundation of China (No. 31571421), Shandong Provincial Natural Science Foundation, China (No. ZR2021MH107), Medical Health Science and Technology Project of Shandong Provincial Health Commission (No. 202002020873, No. 202002021270), Shandong First Medical University (Shandong Academy of Medical Sciences) Youth Science Foundation Cultivation Support Program (No. 202201‐012).

## CONFLICT OF INTEREST STATEMENT

All authors indicate no conflict of interests.

## Data Availability

The data that support the findings of this study are openly available at https://doi.org/10.1111/jcmm.18030.
